# Rare Case of Large Bowel Obstruction due to Plasmacytoid Urothelial Carcinoma

**DOI:** 10.70352/scrj.cr.25-0301

**Published:** 2025-08-26

**Authors:** Kimihiko Nakamura, Toru Inoue, Kazuhiro Ohya

**Affiliations:** 1 Department of Surgery, Kanto Central Hospital of the Mutual Aid Association of Public School Teachers, Tokyo, Japan; 2 Department of Pathology, Kanto Central Hospital of the Mutual Aid Association of Public School Teachers, Tokyo, Japan; 3 Department of Urology, Kanto Central Hospital of the Mutual Aid Association of Public School Teachers, Tokyo, Japan

**Keywords:** large bowel obstruction, extrinsic compression, plasmacytoid urothelial carcinoma, peritoneal dissemination, bladder cancer, diagnostic laparoscopy

## Abstract

**INTRODUCTION:**

The most common etiology of large bowel obstruction (LBO) is colorectal cancer. However, extrinsic compression may occur from cancer of other organs. Plasmacytoid urothelial carcinoma (PUC) is a rare subtype of urothelial carcinoma that can present aggressively as an intraperitoneal spread. This report describes a case of LBO caused by PUC, which initially presented a diagnostic challenge.

**CASE PRESENTATION:**

A 68-year-old woman presented with hematochezia and abdominal pain. She had a history of recurrent bladder cancer, treated with chemotherapy and multiple transurethral resections of bladder tumor (TURBT). The third TURBT revealed invasive papillary urothelial carcinoma (UC; ≥pT2) with partial positivity suggestive of a plasmacytoid or lymphoma-like subtype, while the fourth showed noninvasive UC. CT revealed wall thickening and obstruction of the transverse colon near the hepatic flexure without enlargement of the lymph nodes. Colonoscopy showed luminal narrowing caused by edematous wall thickening, suggesting extrinsic compression. To establish a diagnosis, the patient underwent diagnostic laparoscopy, which revealed that the right side of the transverse colon and the greater omentum were firmly compressed together and immobile, with extensive peritoneal dissemination. An ileo-transverse colon bypass and excisional biopsy of the disseminated nodules were performed, leading to the diagnosis of PUC. Although the patient’s postoperative course was initially satisfactory and oral intake was resumed, duodenal constriction caused by tumor invasion subsequently developed, necessitating the initiation of parenteral nutrition. After undergoing 2 courses of pembrolizumab, pulmonary edema with pneumonia occurred. Despite best supportive care, the patient died 69 days after the operation.

**CONCLUSIONS:**

PUC is a rare and aggressive subtype of bladder cancer that frequently metastasizes, including to the peritoneum, which may lead to LBO. Although colorectal cancer is the most common cause of LBO, considering alternative differential diagnoses and planning appropriate subsequent management are essential in such cases.

## Abbreviations

BC
: bladder cancer
ICI
: immune checkpoint inhibitor
LBO
: large bowel obstruction
PUC
: plasmacytoid urothelial carcinoma
TURBT
: transurethral resection of bladder tumor
UC
: urothelial carcinoma

## INTRODUCTION

LBO is one of the main causes of hospital admission, which is a potentially life-threatening condition that requires prompt diagnosis and management. The most common cause of LBO is colorectal cancer accounting for over 60% of all large bowel obstructions.^1)^ However, the etiology of large bowel obstruction can range from idiopathic and malignant causes to complications resulting from surgery or trauma. Establishing the cause of LBO can be difficult in some cases but is necessary for appropriate treatment.^1,2)^

PUC is a rare and aggressive subtype of UC which possesses a high potential for invasion and distant metastasis. Owing to the loss of E-cadherin, PUC patients tend to present with nodal metastases more often than patients with conventional UC do.^3)^ We report a unique case of LBO in which the initial diagnosis was challenging and was ultimately attributed to PUC.

## CASE PRESENTATION

**Fig. 1:**
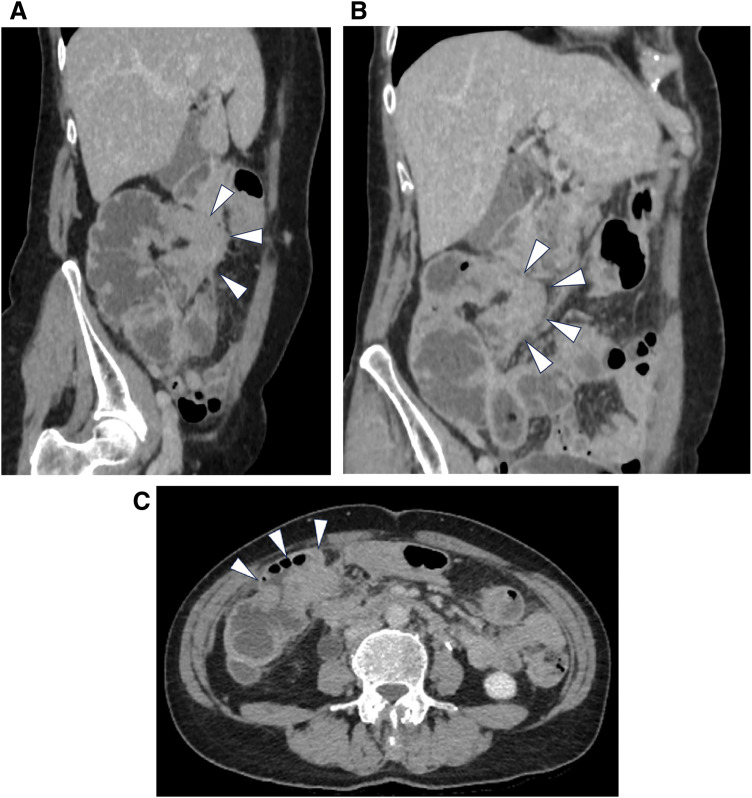
Contrast-enhanced CT of the abdomen. Wall thickening and obstruction of the transverse colon near the hepatic flexure were observed, without associated small intestinal dilation (arrowheads). No lymph node enlargement was noted. (**A**) Coronal section; (**B**) Sagittal section; (**C**) Axial section.

**Fig. 2:**
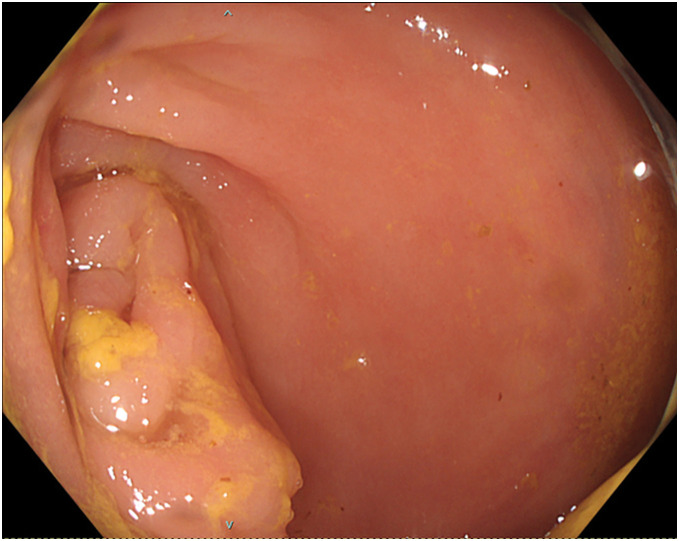
Colonoscopy. Showing luminal narrowing due to wall thickening in the transverse colon, consistent with CT findings; however, visualization of the tumor from the anterior view was difficult.

A 68-year-old woman with a 7-day history of hematochezia and abdominal pain presented to the hospital. She had a 1-year history of deep vein thrombosis controlled with apixaban and a 20-year history of recurrent BC. The patient had undergone 3 courses of chemotherapy (gemcitabine and cisplatin) after 3 rounds of TURBT, which revealed invasive papillary UC of at least pT2, with partial positivity suggestive of either the plasmacytoid or lymphoma-like subtype. Surgical margins were negative, and the resection was deemed curative. She underwent careful follow-up with frequent bladder biopsy and urine cytology after the 4th TURBT, which revealed non-invasive papillary UC. As laboratory testing revealed a D-dimer level of 29.5 µg/mL, a contrast-enhanced CT scan was performed, revealing pulmonary embolism and obstruction of the transverse colon near the hepatic flexure without enlargement of the lymph nodes near the obstruction or apparent dilation of the small intestine (**Fig. 1**). Since her general condition was stable, colonoscopy was performed. Although it was difficult to examine the tumor anteriorly, extrinsic compression was probable, as luminal narrowing caused by edematous wall thickening was observed, and additional brushing cytology was negative for malignancy (**Fig. 2**). Additionally, her tumor marker levels were increased, at 56.2 ng/mL for CEA and 9738.3 U/mL for CA19-9.

**Fig. 3:**
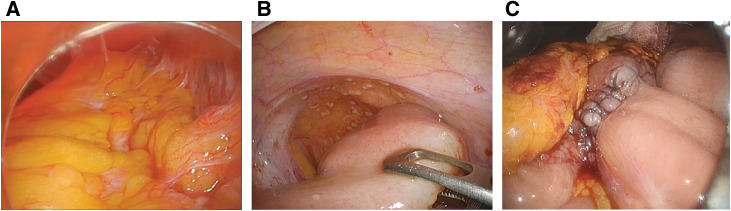
Intraoperative findings. (**A**) The right side of the transverse colon and the greater omentum were firmly adherent and immobile, making partial colectomy difficult. (**B**) Widespread peritoneal dissemination was observed throughout the intraperitoneal cavity. (**C**) Ileo-transverse colon bypass was performed.

At that point, based on the medical history, recurrence of BC was suspected; however, it was considered unlikely given the previous staging. Despite the colonoscopy findings, colon cancer could not be ruled out. For appropriate management of the LBO, a precise diagnosis was essential. The patient therefore underwent diagnostic laparoscopy under general anesthesia in the supine position. A camera port was placed at the umbilicus, and 5-mm ports were inserted in the lower right abdomen and midline of the lower abdomen. Intraoperative findings revealed that the right side of the transverse colon and the greater omentum were firmly compressed together and immobile and that extensive peritoneal dissemination was present. However, there was no direct invasion of the tumor from the bladder. After confirming the absence of small bowel obstruction and considering the patient’s good general condition and quality of life, an ileo-transverse colon bypass was planned instead of a colostomy, as colonic resection was deemed difficult due to the immobility of the tumor (**Fig. 3**). An additional 5-mm port was placed in the left lower abdomen, and the 5-mm port in the right lower abdomen was converted to a 12-mm port. An ileo-transverse colon bypass was then performed, 40 cm proximal to the terminal ileum. The anastomosis was antiperistaltic and created using a 60-mm purple Tri-Staple cartridge (Signia Stapling System; Medtronic, Dublin, Ireland). The entry hole was closed with hand-sewn sutures using a 3-0 V-loc closure device (Medtronic, Dublin, Ireland). Excision biopsy of the disseminated nodules was also performed. The operation time was 206 min and estimated blood loss was 10 g.

**Fig. 4:**
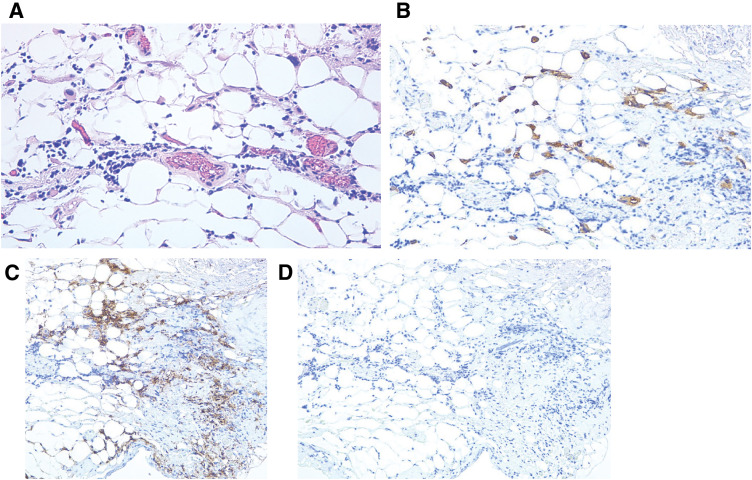
Histopathological examination of the disseminated nodules. (**A**) Hematoxylin and eosin staining shows tumor cells resembling plasma cells with eccentric nuclei and clear cytoplasm (×250); Immunohistochemistry was positive for CK AE1/3 (**B**), CK7 and CD138 (**C**), and negative for E-cadherin (**D**) compatible with plasmacytoid subtype of urothelial carcinoma (×100).

Histopathological examination revealed tumor cells with plasmacytoid morphology, characterized by eccentric nuclei and eosinophilic cytoplasm. Immunohistochemical staining showed positivity for CK AE1/3, CK7, and CD138, and loss of E-cadherin expression, leading to a diagnosis of PUC (**Fig. 4**). Although the patient’s postoperative course was initially satisfactory and oral intake was resumed, duodenal constriction caused by tumor invasion subsequently developed, necessitating the initiation of parenteral nutrition. After undergoing 2 courses of pembrolizumab, the patient suddenly experienced dyspnea, and a CT scan revealed pulmonary edema with pneumonia. The best supportive care was administered, and the patient died 69 days after the operation.

## DISCUSSION

The most frequent etiology of LBO is colorectal cancer, especially in the elderly, but the causes can range from idiopathic and malignant conditions to complications arising from surgery and trauma.^2)^ While the diagnosis of the etiology of LBO is often straightforward when the underlying cause is colorectal cancer, volvulus, or diverticular disease, it can be more challenging in cases with rare causes like in our case. Colonoscopy and CT scans are necessary for identifying the cause of LBO, but diagnostic laparoscopy may be required in case of extrinsic compression. Extrinsic compression of the colon, caused by external factors, can result from a variety of benign and malignant conditions. Examples include large bladder calculi, uterine fibroids, pregnancy, and pelvic malignancies.^1)^ When confined to malignancies of abdominal origin, the colon, ovary, stomach, pancreas, bladder, and endometrium are the most commonly reported primary sites.^4)^ The management of LBO depends on the underlying cause and severity, which posed a significant challenge in our case. Colonic stenting is one of the treatment options for LBO; however, it carries risks such as perforation, stent migration, and potential dissemination of malignancy. In our case, stenting was not recommended for the following 3 reasons: 1. Insertion of a colonic stent for acute angulation, such as the hepatic flexure, is associated with a greater risk of stent erosion or migration; 2. As the diagnosis was not clear before the surgery, placement of a colonic stent could have constrained further therapeutic strategies; and 3. The patient was not in a palliative state, which would otherwise have been an appropriate indication for colonic stent placement.^2)^

In our case, the rare cause of LBO was PUC, a rare and aggressive histologic subtype of BC that accounts for only 1–3% of all BCs. PUC has been included in the WHO classification since 2004 as a subtype of UC.^5)^ Owing to the loss of E-cadherin, PUC exhibits a non-aggregatory spread associated with a greater propensity for extension along the pelvic fascial planes and peritoneal dissemination. Accordingly, PUC patients tend to present with nodal metastases more often than patients with conventional UC do.^3,5)^ The diagnosis, grading, and staging of PUC are mostly the same as those of conventional UC, although PUC patients present with a higher stage. Cystoscopic evaluations, including biopsy and TURBT, are essential for diagnosis and grading. For staging, upper urinary tract imaging, mainly CT urograms and CT scans, is important for metastatic work-up.^6)^ The histological features of PUC are tumor cells resembling plasma cells with eccentric nuclei and eosinophilic or clear cytoplasm infiltrating the bladder wall.^3,5)^ Immunohistochemistry helps further narrow the diagnosis, as the cells express markers of the urothelial lineage, such as GATA3, p63, high-molecular-weight cytokeratin, and uroplakin, along with the plasma cell marker CD138.^5)^ Loss of E-cadherin expression, as mentioned above, facilitates the dissociation of tumor cells, allowing them to migrate individually through tissue planes. This mechanism contributes to peritoneal accumulation, resulting in diffuse peritoneal seeding and, in some cases, omental caking, consistent with our case.^5,6)^ The suitable treatment for PUC is not well-defined because of its rarity, but the therapeutic approach is similar to that for other types of UC. For nonmuscle invasive BC, as the plasmacytoid subtype is categorized as highest risk, radical cystectomy is recommended. For muscle invasive BC, neoadjuvant cisplatin-based chemotherapy is recommended prior to radical surgery, although it is unclear whether patients with PUC experience a survival benefit from neoadjuvant chemotherapy.^7)^ For metastatic disease, cisplatin or carboplatin is recommended if eligible; otherwise, ICIs, such as atezolizmub or pembrolizmab, are recommended as was chosen in this case.^8)^ PUC is associated with a high tumor mutation burden, suggesting that ICIs may be effective in this subtype.^9)^ Although patients with metastatic BC often present with multiple metastatic sites, peritoneal dissemination is reported to be relatively common, occurring in approximately 16% of cases. However, LBO secondary to peritoneal metastasis remains a rare clinical manifestation.^10,11)^ The same applies to PUC, where several cases of small bowel obstruction have been reported, while LBO remains rare.^12,13)^

## CONCLUSIONS

LBO is a major cause of hospital admission but the etiology varies. PUC is a rare and aggressive subtype of BC that frequently metastasizes, including to the peritoneum, which may lead to LBO. This case illustrates a rare finding of a rare subtype of metastatic BC, plasmacytoid, with the development of LBO. It highlights the importance of managing LBO based on its underlying cause, including consideration of diagnoses other than colorectal cancer.
